# Evolving methodology of national tobacco control investment cases

**DOI:** 10.1136/tc-2023-058336

**Published:** 2024-05-02

**Authors:** Rachel Nugent, Brian Hutchinson, Nathan Mann, Carrie Ngongo, Garrison Spencer, Daniel Grafton, Roy Small

**Affiliations:** 1 Department of Global Health, University of Washington, Seattle, Washington, USA; 2 RTI International, Research Triangle Park, North Carolina, USA; 3 United Nations Development Programme, Istanbul, Turkey; 4 HIV, Health and Development Group, United Nations Development Programme, New York, New York, USA

**Keywords:** Economics, Low/Middle income country, Advocacy, Taxation

## Abstract

**Background:**

This article describes an investment case methodology for tobacco control that was applied in 36 countries between 2017 and 2022.

**Methods:**

The WHO Framework Convention on Tobacco Control (FCTC) investment cases compared two scenarios: a base case that calculated the tobacco-attributable mortality, morbidity and economic costs with status quo tobacco control, and an intervention scenario that described changes in those same outcomes from fully implementing and enforcing a variety of proven, evidence-based tobacco control policies and interventions. Health consequences included the tobacco-attributable share of mortality and morbidity from 38 diseases. The healthcare expenditures and the socioeconomic costs from the prevalence of those conditions were combined to calculate the total losses due to tobacco. The monetised benefits of improvements in health resulting from tobacco control implementation were compared with costs of expanding tobacco control to assess returns on investment in each country. An institutional and context analysis assessed the political and economic dimensions of tobacco control in each context.

**Results:**

We applied a rigorous yet flexible methodology in 36 countries over 5 years. The replicable model and framework may be used to inform development of tobacco control cases in countries worldwide.

**Conclusion:**

Investment cases constitute a tool that development partners and advocates have demanded in even greater numbers. The economic argument for tobacco control provided by this set of country-contextualised analyses can be a strong tool for policy change.

**WHAT IS ALREADY KNOWN ON THIS TOPIC**

- Economists have used different models to assess the economic impacts of tobacco use in countries and globally. Results demonstrate economic costs from tobacco use but are not easily compared due to variation in modelling and outcomes measured.

**WHAT THIS STUDY ADDS**

- This study describes a common methodology that was applied in 36 countries to evaluate the economic impacts of tobacco use and the return on investments in tobacco control between 2017 and 2022. While country tailoring allowed for different interventions and methodological adjustments were made over time, the model and process are largely comparable across most of the 36 countries.

**HOW THIS STUDY MIGHT AFFECT RESEARCH, PRACTICE OR POLICY**

- This study, and the others in the collection, provide an economic argument for stronger tobacco control in low-income and middle-income countries.

## Introduction

Tobacco consumption causes disease, premature death, high health costs, economic losses and socioeconomic disparities.^1^ The WHO Framework Convention on Tobacco Control (WHO FCTC) is an international treaty that entered into force in 2005 and currently has 183 member parties. It creates a legal obligation for the parties to implement evidence-based interventions for effective tobacco control. Tobacco control investment cases establish the imperative for countries to act by quantifying the national ‘burden’ of tobacco use, or the total health and economic losses caused by its use. The cases also evaluate the costs and benefits of employing established WHO FCTC tobacco control measures in specific countries, while analysing the policy landscape to assess promising policy opportunities to fulfil WHO FCTC obligations.

This article describes the evolution of an investment case methodology for tobacco control that was used by the WHO FCTC and its partners in 36 countries between 2017 and 2022. The methodology builds upon existing economic models that seek to quantify the costs of tobacco use and benefits of tobacco control. This article details how the investment cases have been applied across countries and how the methods have evolved over time. Some information regarding the WHO FCTC investment case methodology has been previously documented, in online reports and a published article synthesising results from countries in the Americas.^2 3^

### Methods

We developed a credible and consistent—yet flexible—country-specific tobacco control investment case methodology which we applied in three dozen countries. RTI International led the development of the economic modelling and United Nations Development Programme led the institutional context work for the WHO FCTC investment cases. The aim was to produce modelling and contextual assessment that would be generalisable enough to be usable across countries and directly by country stakeholders, and that could evolve with each country’s experience and advances in the academic literature.

We conducted our economic modelling from a modified societal perspective that captured broad socioeconomic impacts attributable to tobacco use, including health-related losses, and social (ie, the value of lives lost due to tobacco use) and productivity losses (ie, absenteeism, presenteeism). We consider the perspective *modified* because we do not capture all external impacts of tobacco use (eg, pollution, deforestation).

Broadly, the economic analyses consisted of two components. In the tradition of cost-of-illness studies, we assessed the burden of tobacco use in each country (ie, calculated total 1-year tobacco-attributable socioeconomic losses). Next, the cases assessed the extent to which tobacco control measures could reduce the burden. In a return-on-investment analysis, over 15 years, we compared two scenarios: a base case in which the 1-year socioeconomic losses are assumed to extend, year over year, with no advances in tobacco control (sometimes called the ‘status quo’ or ‘no action’ scenario), and an intervention scenario showing the outcomes that could be achieved by fully implementing and enforcing evidence-based tobacco control demand reduction measures.

Results from the analysis are reported in Mann *et al* in 2021 US$.^4^ Below, we discuss the data and methods we used from 2017 to 2022, describing instances where our approach or data source(s) changed to align with evolving evidence or best practices. More detailed methods and explanation of the underlying data may be viewed in the online supplemental file 1 and country-specific data used in investment cases may be found in annexes of individual country investment cases that are available online.^2^

10.1136/tc-2023-058336.supp1 Supplementary data

### Identifying the best available data

Data sources for key parameters are listed in table 1. Ideally, data must be credible, nationally representative and recent. For each WHO FCTC investment case, we sought context-specific data to reflect the epidemiological and economic conditions in the country and fill a standardised list of model parameters. These included gross domestic product (GDP), labour force size and employment rates, average salary or wages, average hours worked per day, month and year, excess absenteeism due to smoking, excess presenteeism due to smoking, unsanctioned smoking breaks, healthcare expenditures, the smoking-attributable fraction (SAF) of healthcare expenditures, prevalence of current cigarette smoking, population, life expectancy and death rates.

We worked with government stakeholders to collect, calibrate, and adjust national and locally available data, where available and preferred. To do so, we submitted data collection forms to national health authorities seeking their knowledge of available sources for relevant parameters and often held virtual meetings to follow up and connect with key academic or government authorities.

Some data points were readily available from most—though not all—national governments (eg, employment rates, GDP, healthcare expenditures, population, tobacco use prevalence). Other data were only sometimes forthcoming (eg, average annual salary/wages, annual quit attempt rates among current smokers, price and/or income elasticity of demand measures for tobacco products, rates of use of evidence-based forms of treatment such as nicotine replacement therapy, behavioural therapy), or not or rarely locally available (eg, tobacco-attributable morbidity and mortality, tobacco-related healthcare expenditures and value of a statistical life year (VSLY)).

In cases where no context-specific data were nationally available, with country agreement, we defaulted to use of country-specific data from global models or databases, or regional or income group-specific averages in published online sources.

**Table 1:** Tobacco control investment case data sources

Parameter	Default source
Demographic, epidemiological	
Background mortality	Global Health Observatory Data Repository^5^
Life expectancy, by age and sex	Ibid^5^
Population, by age and sex	The Global Burden of Disease Study^8^
Tobacco-attributable mortality and morbidity	Ibid^8^
Economic	
Average annual salary/wages	ILOSTAT Explorer^31^
Discount rate	Haacker *et al* 32
Employment rate	ILOSTAT Explorer^31^
Gross domestic product	The World Bank Database^33^
Healthcare expenditures, by source	WHO Health Expenditures Database^9^
Healthcare expenditures, smoking-attributable fraction	Goodchild *et al* 7
Value of a statistical life year	Extrapolation methods from Robinson *et al* 11 10; base value of a statistical life estimate from the USA
Tobacco control measures	
Baseline implementation level	WHO Report on the Global Tobacco Epidemic^1^
Costs—planning, implementation and operational	WHO Non-communicable Disease Costing Tool^29^
Impact—non-tax and non-cessation tobacco measures	Levy *et al* 15 as adapted by WHO^14^
Impact—taxation: price and income elasticity of demand	Nargis *et al* 16
Impact—cessation (healthy lifestyle counselling)	Levy *et al* 18
Tobacco use, and cessation rates and strategies	
Smoking prevalence	WHO Report on the Global Tobacco Epidemic^1^
Annual quit attempt rates among current smokers	LMIC averages from GATS and STEPS surveys
Rates of use of evidence-based forms of treatment among smokers who attempt to quit	LMIC averages from GATS and STEPS surveys
Workplace productivity, tobacco-attributable losses	
Excess absenteeism	Troelstra *et al* 12
Excess presenteeism	Baker *et al* 13
Unsanctioned smoking breaks	Berman *et al* 6

GATS, Global Adult Tobacco Survey; LMIC, low-income and middle-income country.

### Estimating tobacco’s health burden

Our model uses country-specific estimates of annual tobacco-related mortality and morbidity from the Global Burden of Disease Study (GBD).^5^ The GBD lists the diseases causally associated with tobacco use (online supplemental file 2).^6^ For each of these causally related diseases, we downloaded GBD estimates of both the total number of deaths or cases, as well as the number of deaths or cases attributable to tobacco use, by sex, 5-year age group and tobacco-related risk factor (smoking, secondhand smoke exposure and smokeless tobacco use). Using these estimates from the GBD data, we derived a population-attributable fraction (PAF) for tobacco use for each sex, age group and tobacco-related risk factor in the data. PAF quantifies the contribution of a specific risk factor of a disease to the total burden of that disease. It is a function of exposure to a specific risk factor and the relative risk of that risk factor, or the ratio of the probability of disease in the exposed population to the probability of disease in the unexposed population. We summed tobacco-attributable mortality and morbidity across disease, sex and age groups to obtain total health impact for each tobacco risk factor (smoking, secondhand smoke exposure and smokeless tobacco use).

10.1136/tc-2023-058336.supp2 Supplementary data

### Estimating tobacco’s socioeconomic burden

The socioeconomic costs of tobacco use fall on households, businesses, public health systems and many other parts of society. Costs include expenditures for smoking-attributable healthcare needs. Most countries lack recently quantified smoking-attributable healthcare expenditures. To estimate healthcare costs in those countries, we followed Goodchild *et al*, who estimated the SAF of healthcare expenditures in 2012 in 152 countries.^7^ We updated Goodchild *et al*’s model, recalculating values using tobacco-attributable death rates from GBD,^8^ and then multiplied the obtained SAF of healthcare expenditures by total health expenditures reported in the WHO Health Expenditures Database^9^ to quantify total healthcare spending attributable to smoking.

Socioeconomic costs consisted of (1) the value of lives lost to tobacco and (2) workplace productivity losses caused by ill-health due to tobacco use. For the former, we monetised future years of life lost using VSLY measures—which reflect individuals’ willingness to pay for changes in life expectancy. We chose VSLY, rather than an economic measure reflecting the productive output of individuals, to ensure we valued all deaths and not just deaths of those participating in the workforce. The VSLY estimate was made following the Reference Case Guidelines for Benefit-Cost Analysis in Global Health and Development^10^ which recommend—in the absence of context-specific VSLY values—extrapolating country-specific VSLY from research in high-income countries. Using a US-based VSL reference,^11^ gross national income (GNI) per capita for income reference (USA) and GNI per capita target (analysis country) values (GNI per capita) and the recommended elasticity of 1.5, we calculated VSL in the target analysis country as follows:

$$VSLTargetCountry=VSLReferenceCountry\times {\left(\frac{IncomeTargetCountry}{IncomeReferenceCountry}\right)}^{\hat{E}lasticity}$$

This produced a population-average VSL value for adults. To convert to VSLY, we divided VSL by undiscounted future life expectancy at the average age of the adult population in that country and multiplied VSLY by the number of life years lost due to tobacco use to estimate the value of tobacco-attributable mortality.

To value workplace productivity losses, we drew on published global estimates comparing productivity impairment of smokers versus non-smokers, including for absenteeism—excess days of work missed—and presenteeism—excess productivity impairment due to health problems caused by tobacco use. For absenteeism, we used a parameter value of 2.9 days of work missed per year due to smoking-related illnesses and for presenteeism a value generating productivity impairment equivalent to about 7.5 days of work lost. For a limited set of initial countries, we also valued unsanctioned smoking breaks (estimates that smokers take around 10 min more time in breaks from work compared with non-smokers).^6 12 13^ Lost time due to smoking was valued by multiplying lost worker time by average wage rates in countries, factoring in the share of the population that was employed.

### Estimating intervention impacts

After estimating the baseline health and economic costs associated with tobacco-related illness, we conducted a counterfactual analysis to determine the potential impact of implementing or expanding key WHO FCTC demand reduction measures. These typically included:

- Tax measures to reduce demand for tobacco products (Article 6).
- Protections from exposure to tobacco smoke in indoor—and where appropriate outdoor—public places (Article 8).
- Regulatory requirements that textual and pictorial health warnings cover 50% or more of the principal display areas of tobacco packaging and that warnings are regularly rotated (Article 11).
- Ensuring that the remainder of the package is devoid of logos, colours, brand images or promotion information, and that only a single standard colour and font style is applied, ‘that is, plain packaging’ (Article 11).
- Use of all available communication tools to promote and strengthen public awareness of tobacco-control issues (Article 12).
- Completely banning tobacco advertising, promotion and sponsorship (Article 13).
- Promoting tobacco cessation through scaling administration of counselling (ie, provision of brief advice to quit tobacco use) at the primary care level within the health system (Article 14).

These measures, as agreed to by parties to the WHO FCTC, are the most cost-effective actions countries can take to reduce the harms from tobacco consumption.^14^ Within the investment cases, with the exception of the tax and cessation measures, the measures’ impact on reducing tobacco use prevalence is from Levy *et al*, as adapted by the WHO for Appendix 3 of the WHO Global Action Plan for the Prevention and Control of Noncommunicable Diseases 2013–2030.^14 15^ For tax measures, where available, price and income elasticities of demand were drawn from country and regional elasticity studies or otherwise from a global modelling study that derived estimates by country income status.^16^ Prevalence elasticities were assumed to be half of price elasticity.^17^ The impact of healthy lifestyle counselling in primary care settings was assessed following methods from Levy *et al*.^18^

Table 2 shows the interventions selected by each country that were included in the investment case analysis. Although all investment case countries are signatories to the WHO FCTC, there is uneven implementation of tobacco control demand reduction measures obligated under the convention. To understand baseline levels of implementation for each measure, we reviewed WHO Global Tobacco Control Reports and elicited updates on the status of implementation from national health authorities and tobacco control authorities.^1^ Target goals for implementation analysed in the implementation scenario were mapped to obligations under the WHO FCTC. The incremental impact of implementing tobacco control demand reduction measures when considering different baselines was sourced from the United Nations Interagency OneHealth Tool.^19^

**Table 2:** FCTC tobacco control policies and custom interventions in FCTC investment cases

	Warning labels (FCTC Art. 11)	Bans on advertising (FCTC Art. 11/13)	Raise cigarette taxes (FCTC Art. 6)	Protect people from tobacco smoke (FCTC Art. 8)	Mass media campaigns (FCTC Art. 12)	Plain packaging (FCTC Art. 11 Guidelines/Art. 11)	Cessation support: brief advice to quit tobacco (FCTC Art. 14)
Armenia							
Bhutan							
Burkina Faso							
Cabo Verde							
Cambodia							
Chad							
Colombia							
Costa Rica							
Egypt							
El Salvador							
Eswatini							
Fiji							
Georgia							
Ghana							
Iran							
Jordan							
Laos							
Lebanon							
Madagascar							
Mongolia							
Montenegro							
Mozambique							
Myanmar							
Nepal							
Pakistan							
Panama							
Samoa							
Serbia							
Sierra Leone							
Sri Lanka							
Suriname							
Tanzania							
Timor-Leste							
Tunisia							
Vanuatu							
Zambia							

Cells highlighted in green indicate that the country was meeting FCTC obligations for the tobacco control measure at the time of the investment case analysis. Thus, no modelling of these measures was included in the analysis.

FCTC, Framework Convention on Tobacco Control.

Some countries requested analysis of additional actions they would like to consider (eg, restriction on tobacco cultivation, banning single stick cigarettes). Others requested analysis of additional outcomes that may result from tobacco control implementation (eg, the implications of increases in cigarette taxation for government revenue or population equity). Where countries indicated preferences and data allowed, we conducted these custom analyses. Methods for these custom analyses may be found in country reports available online.^2^ Results from a commonly requested custom analysis—equity implications of tobacco taxation—are reported by Spencer *et al*.^20^

The return-on-investment analysis compared socioeconomic losses in the ‘no action’ and intervention scenarios. In doing so, it compared differences in the expected health outcomes (ie, deaths, disability-adjusted life years) and socioeconomic outcomes (ie, tobacco-attributable healthcare expenditures, workplace productivity losses and value of losses in life due to tobacco use). In the analyses start years, we established the PAF of tobacco-attributable mortality and morbidity and the SAF for healthcare expenditures—as described above for the baseline, ‘no action’ scenario. Over the 15-year time horizon of the analysis, we adjusted the PAF and SAF for the intervention scenario based on the year-over-year changes in adult smoking prevalence and assumed commensurate drops in tobacco-attributable mortality, morbidity, healthcare expenditures and workplace productivity losses. Economic costs and benefits in future years were discounted and compared to produce a measure of return on investment for individual tobacco control measures, and for the collective impact of the measures. Mann *et al* provides a full accounting of these results and places them in the context of country demographic, economic and health conditions.^4^

### Understanding the institutional context

The economic analysis was complemented by an institutional and context analysis (ICA) to assess the political and economic dimensions of tobacco control in each country. The ICA sought to uncover promising policy pathways to WHO FCTC implementation and enforcement in line with national circumstances. It included three main steps: (1) desk-based review of country context, including on how tobacco interacts with sustainable development priorities; (2) landscape analysis and key informant interviews to assess factors related to planning, coordination, financing and tobacco industry interference in policymaking; and (3) documentation of results in an ICA report which includes a clear plan for increasing political space for tobacco control. The ICA informs investment case recommendations related to governance and financing. It also supports advocacy and communications.

## Results

Mann *et al* provides a full accounting of these results and places them in the context of country demographic, economic and health conditions.^4^

### Improving the model

We developed the WHO FCTC investment case methodology in 2017 and have since applied it in 36 countries. Each country has provided the opportunity to learn and adapt the WHO FCTC investment case approach, both to align with country priorities and to strengthen the model and its results. We grappled with how to model extremely low and high levels of tobacco prevalence; analyse the cost and effect of interventions for which little evidence was available (eg, single stick bans); and incorporate weak evidence about some of the key country information which required negotiation with public health officials. We have been fortunate to receive inputs and suggestions from country-based economic experts and worked with them to adapt our model to the specifics of the local policy environment.

While the foundation remained stable, we adapted the method over time to stay current with best-practice guidelines for economic evaluations of health programmes and policies; respond to country demand for custom interventions; incorporate new data sources; and recognise diverse cultural and macroeconomic conditions. We made several methodological improvements that were applied in the later WHO FCTC investment cases. These followed a top-to-bottom review of the model considering advances in the investment case and economic evaluation literature in the intervening 5 years.

Four major adjustments in our economic evaluation methods were made. First, in 2020, we shifted to using VSLY to measure the economic value of extensions in life due to quitting tobacco use. VSLY measures the valuation people place on a reduced risk of mortality in a year. We had been using the ‘full income’ approach to life year valuation, following the method introduced by the Lancet Commission on Investing in Health.^21^ This measures the value of a year of life expectancy gained and is intended to be a more intuitively appealing method to estimate the economic value of healthy life years. However, following its publication in 2013, it has received some critique and has not been widely adopted.^22^ We adopted the VSLY measure recommended by the Reference Case Guidelines for Benefit-Cost Analysis in Global Health and Development, which is intended to standardise methods, establish best practices and provide default values for key parameters for researchers conducting economic evaluations.^10^ The general effect has been to reduce our benefit estimates in the intervention scenario; but this did not significantly alter the final results of recent cases.

Second, we explored the scope and validity of intervention costs, which were taken from the WHO Non-communicable Disease Costing Tool released in 2011. Updating the tool to estimate costs in later years is an easy adaptation^23^—which we applied. However, we wished to better understand some of the costs that governments would pay to strengthen tobacco control and augment those costs with new data. Specifically, these included administration and enforcement of tax and regulatory measures and costs of cessation-based measures. For instance, for promoting tobacco cessation through healthy lifestyle counselling, we added training costs for health providers, linked the cost of outpatient visits to the length of time to provide brief quitting advice to tobacco users and scaled costs on expectations of how many users health providers would encounter in a given year.

Third, to better clarify and communicate economic costs of tobacco use, we shifted our language from direct and indirect costs to, respectively, healthcare costs, workplace costs and the cost of lost human life or mortality costs. We updated the data used to inform estimates of absenteeism and presenteeism in the workplace as new evidence emerged. We also removed smoking breaks as part of the productivity loss from tobacco since there is heterogeneity across countries in how smoking is handled in the workplace.

Finally, in the early investment cases, we applied impact sizes of the tobacco control demand reduction measures—drawn from WHO’s Appendix 3— as absolute reductions in smoking prevalence against countries’ baseline smoking rates.^14^ But, with current smoking rates as low as 5% in some countries, applying large absolute reductions became untenable. Thus, we shifted to applying impact sizes as relative reductions in smoking prevalence. Using this method, we converted the WHO’s absolute impact sizes to relative terms using a 25% representative-level smoking rate (ie, in a country with an initial current smoking rate of 20%, where before a 4% absolute reduction in smoking prevalence reduced smoking rates to 16%, now smoking rates were reduced to 16.8%, a 16% relative reduction in smoking prevalence). This change was in better accordance with Levy *et al*—the source of the WHO’s impact sizes. It uses relative reductions by applying absolute reductions to ‘the 25% initial smoking prevalence as a conservative estimate of the initial rates during the time period when most evaluation studies were conducted’.^15^

## Discussion

Investment case methodology varies across global health topics and over time, as new models have been developed and new data become available that allow more comprehensive and country-tailored analysis. The WHO FCTC investment cases discussed in this series advance the field in several ways. Notably, they offer comparable results from a demand-driven process of strengthening tobacco control. The large number of FCTC countries that have not yet undertaken an investment case now have access to a large body of economic results and can monitor the subsequent gains in tobacco control across 36 countries—assuming they transpire—as time passes.

In applying a single method across 36 countries, the WHO FCTC investment cases provide an extensive examination of the economic impacts of a single health risk factor. The cases help fill evidence gaps highlighted by a 2022 systematic review of economic evaluations of tobacco control interventions in low-income and middle-income countries (LMICs).^24^ The review identified 20 studies between 1994 and 2020 worthy of inclusion, but most examined the cost-effectiveness of only single or a few interventions, few used a societal perspective, and diverse methods create a need for standard reporting guidelines.

The FCTC cases create an opportunity for comparison of results across countries that reveals subtle and not-so-subtle barriers and opportunities to achieve better tobacco control. For instance, consumer choices for tobacco products such as bidis, waterpipes or chewing tobacco create the need for additional surveillance methods and policy flexibility. Countries such as Jordan and Egypt, where a large share of tobacco use occurs socially in cafes via waterpipes, call for a discussion of the sociological aspects of tobacco use and how societal norms influence tobacco consumption behaviour. Country comparisons enabled by a common modelling approach also reveal issues whose significance becomes clear in repeated occurrences. An example is the way in which the myth of cross-border smuggling pervades country discussion of tobacco tax increases. This challenge to tax increases was raised early and often in the investment case process—clearly driven by an industry campaign to undermine the effectiveness of tobacco taxation. With the ability to examine the issue across a wide range of country conditions—such as proximity to tobacco-exporting countries, comparative taxing regimes, enforcement intensity, and other factors that influence local availability and prices of tobacco—the WHO FCTC investment cases revealed that cross-border smuggling is not pervasive and generally has a minor impact on domestic availability of cigarettes, thereby strengthening the case for tax increases.

Second, while tobacco control advocacy has a long and strong history, there has not been a concerted effort to highlight the economic arguments for tobacco control since the World Bank effort from the late 1990s.^25^ That effort—like this one—produced a compelling array of economic arguments for stronger tobacco control including pointing out the centrality of tobacco taxation to a comprehensive tobacco control programme. The WHO FCTC investment case collection re-examines and renews the arguments raised 20 years ago, and adds others derived from the detailed analyses from three dozen countries, that garner the attention of finance and economic officials. The cases repeatedly demonstrate that strong tobacco control will augment GDP growth by improving productivity, reducing medical expenditure and averting early loss of life.

Third, by conducting dozens of investment cases across all regions of the world in a compressed period, the WHO FCTC investment case collection intensifies and energises the case for tobacco control globally. The effect is to galvanise multisectoral actors around the potential to advocate for tobacco control. The methods used for each investment case—the process, data, modelling and outputs—are largely common across the 36 countries. This provides a common language and set of actions for countries, differing only in their starting point and policy priorities.

The investment cases serve as a shot in the arm for tobacco control efforts, but the existing methodology does not meet every need that countries have raised. First, new research based in LMICs is needed to reduce the reliance on dated and US-based literature. Gaps remain in the ability to model outcomes of some policy approaches that sit outside the MPOWER package. For instance, some countries have shown interest in quit lines which are extensively used in high-income countries to provide support and counselling to tobacco users. Yet, there are no documented data on how well such tools (or modern versions such as short messaging service) work in LMICs. Another recent focus of tobacco control in many countries is e-cigarettes and vaping. There is controversy over the health risks, and yet, there is no consensus on how those products should be handled in LMIC tobacco control programmes. These and other gaps related to policy design and evidence for impact are only answerable with adequate data about consumption, pricing, and tobacco-related behaviours and updated country-based research that can also reduce the need to rely on official country reports.

As tobacco companies will continue to try and build their markets, countries will continue to innovate in their tobacco control efforts with vigorous policies including adaptations of existing approaches (eg, new labelling innovations and supply-side measures). With the evolution of ways to eliminate tobacco, there will need to be new modelling that reflects the health and economic impacts of such moves.

### Limitations

Even with model enhancements over time, we recognise significant gaps exist in understanding the health and economic impact of tobacco use. First, we relied on modelled tobacco-attributable mortality and morbidity estimates from the GBD. While not without criticism,^26–28^ GBD remains the foremost modelling effort seeking to estimate the global burden caused by diseases and risk factors. Still, future national-level analyses would benefit from health surveillance system data tracked, reported and owned by national institutions. Second, our intervention effects were drawn from studies conducted in areas where manufactured cigarette use is most prevalent. The evidence base for non-cigarette tobacco—the burden and interventions—such as hookah (common especially in Middle Eastern countries) and bidis (especially common in South Asia) is less developed. National governments would benefit from more detailed investigation in areas where these forms of tobacco use dominate. Third, our assessment of tobacco-related healthcare expenditures is based on a crude, top-down approach of applying an SAF to total health expenditures. An SAF that is too low will underestimate tobacco-related healthcare expenditures, while an SAF that is too high will overestimate tobacco-related healthcare expenditures. We performed a rudimentary sensitivity analysis by adding a lower and upper bound to our analysis. We recognise that it would be more precise to build our assessment from data on disease-specific expenditures. Fourth, our estimates of absenteeism and presenteeism are largely based on studied outcomes in high-income countries because few studies have been conducted on health-related productivity losses in LMICs. While our estimates are the best available data, they may not be representative of contexts where labour rules and safety nets differ. Fifth, in working with the officials in each country that were responsible for tobacco control, we were obliged to accept their perspective on choice of interventions to be analysed and their assessment of the effective coverage of pre-existing interventions and policies. Finally, we recognise the imprecision and uncertainty regarding the health benefits and economic costs of implementing tobacco interventions and policies. Intervention costing is an inexact science as many countries do not calculate resource expenditure by disease or risk factor. Our estimates were based on the financial costs of implementation that were produced by the WHO in 2012^29^ and are being updated. These costs may not reflect the current landscape and environment in each country. Nonetheless, quantitative estimates can be valuable for policymakers, especially when they use consistent modelling methods and produce consistently similar results.

### Conclusion

The ultimate judge of the WHO FCTC investment cases is the demonstrated impact they have on the ground. The investment cases are requested by countries that are assessed to have the conditions necessary to advance the tobacco control agenda. The cases are scrutinised by public health authorities and others who wish to promote—and perhaps some who would wish to block—progress on that agenda. If there is widespread action to implement and scale the implementation of the WHO FCTC-recommended interventions in those countries that requested investment cases, there is a prima facie argument that the methods and results are credible, in addition to being generalisable and comprehensive. Small *et al* in this collection describes the evidence to date on how WHO FCTC investment cases have been used and the policy results that might be attributable to them across multiple countries, and Mann *et al* report results for 21 countries.^4 30^

## Data Availability

Data used in the study is available upon reasonable request from BH (bhutchinson@rti.org).
