# Supplementary material for: Evolving methodology of national tobacco control investment cases
Source: Tob Control. 2024 May 2;33(Suppl 1):s10–6. doi: 10.1136/tc-2023-058336 (PMC11103330; doi:10.1136/tc-2023-058336)
Supplement: Supplementary data [file tc-2023-058336supp001.pdf]

Supplemental File 1

Contents

1. Overview of the Tobacco Control Investment Case Economic Model 3
1.1 Cost-of-Illness analysis – estimating the socio-economic burden of tobacco use 4
1.2 Return-on-investment analysis 4
2. Assess tobacco-attributable mortality and morbidity 6
3. Estimate socio-economic losses due to tobacco use 6
3.1 Healthcare costs 6
3.2 Cost of lost human life (social cost) 7
3.3 Workplace productivity losses 7
4. Modeling the Impact of Tobacco Control Measures 8
4.1 The effect of non-tax and non-cessation measures 8
4.1.1 Assessment of baseline levels of implementation relative to target goals 9
4.1.2 Effect sizes of select interventions based on intensity level 10
4.2 The impact of tax measures 11
4.3 The impact of brief advice to quit tobacco use at the primary care level 12
4.4 Combined Impact: Effect Size of Implementing Multiple Policies Simultaneously 13
4.4 Modelling the impact of the measures on health and socio-economic outcomes 13
5. The Financial Cost of Implementing and Enforcing Tobacco Control Measures 14
6. Return on Investment (ROI) 14
7. References 15

1. Overview of the Tobacco Control Investment Case Economic Model

RTI International's Tobacco Control Investment Case Economic Model facilitates two types of health-economic analyses: 1) A cost-of-illness studies analysis that assesses the total socio-economic burden caused by tobacco use in countries worldwide and 2) a return-on investment analysis that assesses the costs and benefits of fully implementing and enforcing key tobacco control demand reduction measures.

Broadly, Figure 1 outlines the primary methodological steps required to perform the analyses. The first two methodological steps are pertinent to the **COI analysis** while steps 3-5 are relevant to the **return-on-investment** analysis.

- **Step 1**: Assess current tobacco-attributable mortality and morbidity in the country.
- **Step 2**: Estimate current total socio-economic costs of tobacco use in the country.
- **Step 3**: Estimate the extent to which tobacco control demand reduction measures can reduce tobacco use prevalence, disease morbidity, and socio-economic losses.
- **Step 4**: Estimate the financial costs (i.e., costs paid by government) to implement and enforce tobacco control demand reduction measures.
- **Step 5**: Quantify the Return on Investment (ROI) for the tobacco control measures modeled, both individually and collectively, over a 15-year period.

Figure 1. Model overview

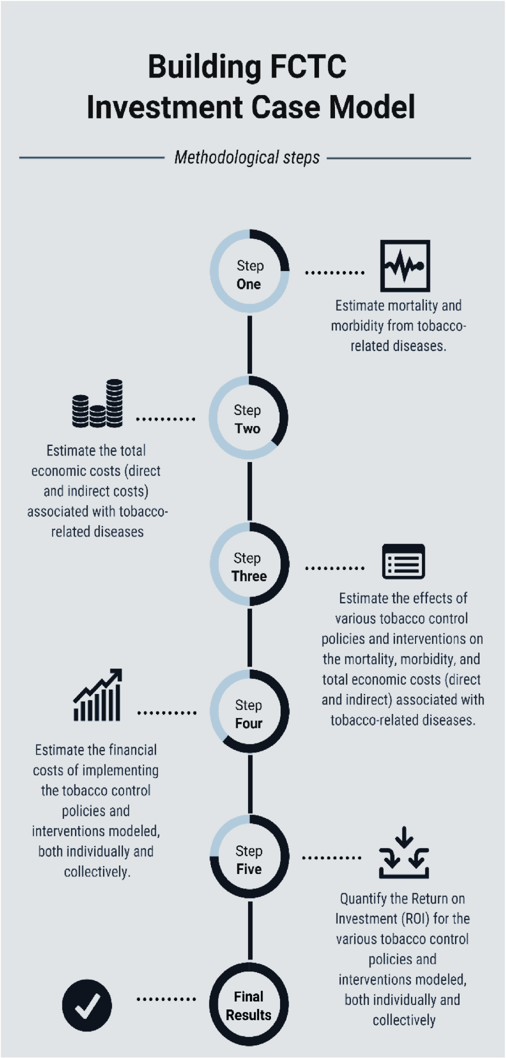

1.1 Cost-of-Illness analysis – estimating the socio-economic burden of tobacco use

The **Cost-of-Illness** (current burden) component of the Tobacco Control Investment Case summarizes tobacco-attributable mortality and morbidity as well as the total socio-economic cost of tobacco use (direct and indirect costs) in each country using the most recent data available. The current burden is meant to provide a one-year snapshot or summary of losses attributable to tobacco use.

Step 1: Assess tobacco-attributable mortality and morbidity. From the Institute for Health Metrics and Evaluation's Global Burden of Disease Study, we obtained estimates of tobacco-attributable mortality and morbidity for four tobacco use risk factors: smoking, secondhand smoke exposure, smokeless tobacco use, and tobacco (which is the combined effect of smoking, secondhand smoke exposure, and smokeless tobacco use).

Step 2: Estimate the total socio-economic costs of tobacco use. The model estimates total costs of tobacco use. Total costs are broken down into.

- **Healthcare expenditures.** Tobacco-attributable healthcare expenditures include those covered by public (government-paid), and private (insurance and out-of-pocket) payers.
- **Lost value of human life**. An estimate of the societal value lost due to early death.
- **Workplace productivity losses**. Tobacco-attributable labor productivity losses due to health impairment caused by tobacco use, including: (1) excess absenteeism – working days missed per year due to smoking, (2) excess presenteeism – the estimated loss of productivity among active smokers at work due to smoking-attributable health impairment and disability; (3) time lost due to unsanctioned smoking breaks above and beyond the breaks that workers are entitled to during the workday (note: consideration of this type of loss was removed after the initial set of investment cases due to heterogeneous handling of smoking in the workplace worldwide).

Data inputs for the current burden estimates come from a variety of data sources that are publicly available on the Internet as well as published peer-reviewed papers and studies. Country-specific data inputs and parameter estimates are also obtained directly from country partners. We estimate the current burden for a single year using the most recent data available.

1.2 Return-on-investment analysis

In a return-on-investment analysis, over 15 years, the model compares two scenarios: a base case in which, with no advances in tobacco control, the one-year socio-economic losses from the current burden component are assumed to extend, year-over-year (sometimes called the "status quo" or "no action" scenario), and an intervention scenario showing the outcomes that could be achieved by fully implementing and enforcing evidence-based tobacco control demand reduction measures.

The model:

Nugent R, *et al. Tob Control* 2024; 33:10–16. doi: 10.1136/tc-2023-058336

1. In the status quo scenario, extends the annual results from the current burden (described in Section 1.1 above) to each of the 15 years for the model period. This means that the current burden will be repeated for each of the 15 years in the model period.
2. Estimates the extent to which tobacco control demand reduction measures reduce tobacco use prevalence over a 15-year period (see section 5).
3. Using a population attributable fraction (PAF) approach, estimates reductions in tobacco-attributable mortality and morbidity, and socio-economic costs (direct and indirect) of tobacco use for the intervention scenario (i.e., in the PAF approach, reductions are commensurate to the drop in tobacco use prevalence). The differences in outcomes between the intervention scenario and the status quo scenario are the savings generated by tobacco control policies and interventions.
4. Estimates the financial costs of implementing the policies/interventions analyzed, individually and collectively using the WHO NCD Costing Tool.
5. Calculates the Return on Investment (ROI) for the policies/interventions analyzed, individually and collectively (methodological **Step 5**).

Table 1 summarizes the data inputs informing the Tobacco Control Investment Case Economic Model and default data sources used if no data was available from national surveys, reports, etc.

Table 1. Key data and default sources

Parameter	Default source
**Demographic, epidemiological**	
Background mortality	Global Health Observatory Data Repository[1]
Life expectancy, by age and sex	Ibid
Population, by age and sex	IHME, Global Burden of Disease [2]
Tobacco-attributable mortality and morbidity (lives lost, years of life lost, years lived with disability, DALYs)	Ibid
**Economic**	
Average annual salary/wages	ILOSTAT Explorer [3]
Discount rate	Haacker et al. (2020)[4]
Employment rate	ILOSTAT Explorer [5]
Gross domestic product (GDP)	The World Bank Database [6](1)
Healthcare expenditures, by source	WHO Health Expenditures Database [7]
Healthcare expenditures, smoking-attributable fraction	Goodchild et al. (2018) [8]
Value of a statistical life year (VSLY)	Extrapolation methods from Robinson et al (2019) [9]; base VSL estimate from United States [10]
**Tobacco control measures**	
Baseline implementation level	WHO Report on the Global Tobacco Epidemic (2021) [11]
Costs - Planning, implementation, and operational	WHO NCD Costing Tool [12]
Impact - Non-tax and non-cessation tobacco measures	Levy et al (2018) [13] as adapted by WHO [14]
Impact - Taxation - Price and income elasticity of demand	Nargis et al (2020) [15]
Impact - Cessation (healthy lifestyle counselling)	Levy et al (2010) [16]
**Tobacco use, and cessation rates and strategies**	

Smoking prevalence	WHO Report on the Global Tobacco Epidemic (2021) [11]
Annual quit attempt rates among current smokers	LMIC averages from GATS and STEPS surveys (compiled by authors)
Rates of use of evidence-based forms of treatment among smokers who attempt to quit	Ibid.
**Workplace productivity, tobacco-attributable losses**	
Excess absenteeism	Troelstra et al (2019) [17]
Excess presenteeism	Baker et al (2017) [18]
Excess smoking breaks	Berman et al (2014) ([19]

2. Assess tobacco-attributable mortality and morbidity

Tobacco-attributable mortality and morbidity for each disease (D), sex (S), and 5-year age group (A) is from IHME's Global Burden of Disease (GBD) study. GBD is led by the Institute for Health Metrics and Evaluation (IHME) at the University of Washington. Data are collected and analyzed by a consortium of more than 2,300 researchers in more than 130 countries.

GBD data that is available freely online. For use in our model, mortality and morbidity data by country was downloaded by sex **S**, disease **D,** 5-year age group **A**, and risk factor **R** (smoking, secondhand smoke exposure, and smokeless tobacco use).

The GBD data accounts for 38 diseases that have strong evidence supporting a causal link with smoking: age-related macular degeneration Alzheimer's Disease and other dementias; aortic aneurysm; atrial fibrillation and flutter; bladder cancer; breast cancer; cervical cancer; chronic obstructive pulmonary disorder; colon and rectum cancer; diabetes mellitus type 2; gallbladder and biliary diseases; intracerebral hemorrhage; ischemic heart disease; ischemic stroke; kidney cancer; larynx cancer; lip and oral cavity cancer; leukemia; liver cancer; lower respiratory infections; multiple sclerosis; nasopharynx cancer; esophageal cancer; other pharynx cancer; otitis media; pancreatic cancer; peptic ulcer disease; peripheral artery disease; prostate cancer; rheumatoid arthritis; stomach cancer; subarachnoid hemorrhage; tracheal, and bronchus, and lung cancer.

3. Estimate socio-economic losses due to tobacco use

3.1 Healthcare costs

Within the investment case modelling, healthcare expenditures attributable to tobacco use (e.g., hospitalization costs, physician costs, medication costs) which may be covered by public (i.e., government) or private payers (e.g., insurance, individuals out-of-pocket).

Total healthcare expenditures, by payer type, were obtained from the *WHO Global Health-care Expenditure Database*. The proportion of health-care costs attributable to smoking was obtained using updated data employing Goodchild and colleagues' (2018) formula for estimating smoking attributable fraction (SAF) of health-care expenditures [8].

$$\textbf{Tobacco-attributable healthcare expenditures} = \text{Total Healthcare Expenditures} \times SAF \text{ of healthcare expenditures}$$

To calculate the share of smoking-attributable health-care expenditures borne by public, non-profit, and private entities, it was assumed that each entity incurred smoking-attributable health-care costs in equal proportion to the entity's contribution to total health expenditure.

3.2 Cost of lost human life (social cost)

The cost of tobacco-attributable mortality accrues when tobacco use causes early mortality, eliminating the unique economic and social contributions that an individual would have provided in their remaining years of life.

We monetized future years of life lost using value of a statistical life year (VSLY) measures—which reflect individuals' willingness to pay for changes in life expectancy. We chose VSLY, rather than an economic measure reflecting the productive output of individuals, to ensure we valued all deaths and not just deaths of those participating in the workforce. The VSLY estimate was used and obtained following the Reference Case Guidelines for Benefit-Cost Analysis in Global Health's [9] which recommend to—in the absence of context-specific VSLY values—extrapolate country-specific VSLY from research in high income countries. Using a US-based VSL reference [10], GNI per capita for income reference (US) and GNI per capita target (analysis country) values (GNI per capita), and the recommended elasticity of 1.5, we calculated VSL in the target analysis country as follows.

$$VSL\ TargetCountry = VSL\ ReferenceCountry * \left(\frac{IncomeTargetCountry}{IncomeReferenceCountry}\right)^{\wedge Elasticity}$$

This produced a population-average VSL value for adults. To convert to VSLY, we divided VSL by undiscounted future life expectancy at the average age of the adult population in that country and then, we multiplied VSLY by the number of life years lost (calculated using age at death and age-specific life table data on the year-to-year probability of survival year to year).

In analyses, the model assigns the monetized value of future years of life lost to the year in which the death occurred. It discounts the value obtained in future years using income-group-specific discount rates (four percent for middle income countries or five percent for low-income countries).[4]

3.3 Workplace productivity losses

To value workplace productivity losses, we drew on published global estimates comparing productivity impairment of smokers versus nonsmokers, including for absenteeism—excess days of work missed —and presenteeism—excess productivity impairment due to health problems caused by tobacco use. For absenteeism, we used a parameter value of 2.9 days of work missed work per year due to smoking-related illnesses [17]. Presenteeism losses are obtained similarly, under research that shows that smokers in China, the United States, and five European countries experience about 22% more impairment at work because of health problems compared to never-smokers—losses equivalent to

Nugent R, et al. Tob Control 2024; 33:10–16. doi: 10.1136/tc-2023-058336

about 7.5 days of work[18]. For a limited set of initial countries, we also valued unsanctioned smoking breaks (estimates that smokers take around 10 minutes more time in breaks from work compared to nonsmokers).[19] Lost time due to smoking was valued by multiplying lost worker time by average wage rates in countries, factoring in the share of the population that was employed.

4. Modeling the Impact of Tobacco Control Measures

Investment cases examined the impact of several key tobacco control demand reduction measures:

- Increasing **taxes** on tobacco products to reduce the demand for tobacco.
    - Target Goal: Comprehensive, well-enforced bans on smoking in all public indoor and outdoor spaces (e.g., health-care, educational, governmental, and other facilities; pubs, bars, and cafes; restaurants; private offices and workplaces; public transport).
- Protecting populations from exposure to tobacco smoke by banning smoking in public places (i.e. **smoke-free air law**)
    - Target Goal: Comprehensive, well-enforced bans on smoking in all public indoor and outdoor spaces (e.g., health-care, educational, governmental, and other facilities; pubs, bars, and cafes; restaurants; private offices and workplaces; public transport).
- Regulating packaging and labelling of tobacco products by mandating that 1**) large graphic warning labels** are displayed on tobacco products and 2) the remainder of the packaging is required to be neutral colors and free of branding (i.e. **plain packaging**)
    - Target goals: Rotating, graphic warning labels that cover 50 percent or greater of the principal display areas. Plain (neutral color) packaging of tobacco products, sans logos, brand images, and promotional information.
- Implementing national-scale education, communication, training and **public awareness campaigns through mass media**;
    - Target goal: A mass media campaign that is part of a comprehensive tobacco control program; is researched and tested with a target audience; includes a media planning and buying process; airs on radio/TV; achieves coverage with journalists, and; has an evaluation component.
- Enforcing a comprehensive ban on tobacco advertising, promotion and sponsorship (**TAPS bans**); and
    - Target goal: Comprehensive, well-enforced bans on all forms of direct (e.g., television, radio) and indirect (e.g., product placement in TV or film, free distribution, promotional discounts) forms of APS.
- Promoting cessation of tobacco use through scale up of **healthy lifestyle counselling** within health systems.
    - Target goal: Train primary healthcare providers to identify tobacco users and to provide tobacco cessation advice; scale the provision of tobacco cessation services at the primary care level.

4.1 The effect of non-tax and non-cessation measures

The effect of several key tobacco control measures on tobacco use prevalence rates has been evaluated in published studies, mainly in high income country contexts. WHO, in collaboration with experts, has published estimates that weigh published literature and adapt estimates for contexts worldwide (see **Table 2**). These country-invariant estimates are published in the Tobacco Use Technical Brief of Appendix 3 of the *WHO Global Action Plan for the Prevention and Control of Non-communicable Diseases 2013-2030*.

Table 2. Effect Sizes for select Tobacco-control measures

Demand reduction measure	Absolute reduction (%) in tobacco use prevalence if target goals are reached [a]	Original source of the effect size or underlying methods to calculate the effect size in academic literature
Smoke Free air laws	4%	Levy et al. (2018)
Graphic warning labels	4% [b]	Levy et al. (2018)
Plain packaging	1% [b]	Chipty et al (2016)
Mass media anti-tobacco campaign	3.8%	Levy et al. (2018)
TAPS bans	10%	Levy et al. (2018)

Ultimately, we converted the absolute reductions in prevalence in **Table 2** to relative reductions using a 25 percent representative-level smoking rate (i.e., in a country with an initial current smoking rate of 20 percent, where before a four percent absolute reduction in smoking prevalence reduced smoking rates to 16 percent, now smoking rates were reduced to 16.8 percent). We used the 25 percent representative level in accordance with the original source of estimates, Levy et al (2018), who note "the 25% initial smoking prevalence as a conservative estimate of the initial rates during the time period when most evaluation studies were conducted".[13]

Except for taxes—the impact of which is dependent on the timing of increases in tax rates (see below)—and the brief cessation advice intervention—the impact of which is guided by rates of training for primary health-care providers—the full impact of the demand reduction policy measures was phased in over a five-year period. The phase-in period followed WHO assumptions that two years of planning and development are required before policies are up and running, followed by three years of partial implementation that are reflective of the time that is needed to roll out policies, and work up to full implementation and enforcement.[12]

4.1.1 Assessment of baseline levels of implementation relative to target goals

Estimates in Table 2 are presented as the expected *absolute* reduction (%) in smoking prevalence that would result from moving from having no provision in place in the status quo, to a scenario in which the provision is fully implemented (i.e., reaches the target goal described above).

Nugent R, *et al. Tob Control* 2024; 33:10–16. doi: 10.1136/tc-2023-058336

However, a country may have gradations of a measure in effect. For instance, it may have a smoke-free air law that bans smoking in workplaces and at educational institutions, but not in other public spaces. Or, it may have a law that bans smoking in all public places, but there may be low compliance with the law, meaning the country is unlikely to receive the full expected effect on the prevalence of smoking.

To take this into account, the investment case modelling assessed the "intensity level" of specific policy measures, with levels corresponding with those designated in Global Tobacco Control Reports (GTCR) (see **Figure 2).**[1]

Figure 2. Intensity Levels of Select Tobacco-Control Measures

SMOKE-FREE POLICIES: POLICIES ON SMOKE-FREE ENVIRONMENTS

	Data not reported/not categorized
	Complete absence of ban, or up to two public places completely smoke-free
	Three to five public places completely smoke-free
	Six to seven public places completely smoke-free
	All public places completely smoke-free (or at least 90% of the population covered by complete subnational legislation)

HEALTH WARNINGS: HEALTH WARNINGS ON CIGARETTE PACKAGES

	Data not reported
	No warnings or small warnings
	Medium size warnings missing some appropriate characteristics OR large warnings missing many characteristics
	Medium size warnings with all appropriate characteristics OR large warnings missing some appropriate characteristics
	Large warnings with all appropriate characteristics

MASS MEDIA: ANTI-TOBACCO CAMPAIGNS

	Data not reported
	No national campaign conducted between July 2014 and June 2016 with duration of at least three weeks
	National campaign conducted with one to four appropriate characteristics
	National campaign conducted with five to six appropriate characteristics, or with seven characteristics excluding airing on television and/or radio
	National campaign conducted with at least seven appropriate characteristics including airing on television and/or radio

ADVERTISING BANS: BANS ON ADVERTISING, PROMOTION AND SPONSORSHIP

	Data not reported
	Complete absence of ban, or ban that does not cover national television, radio and print media
	Ban on national television, radio and print media only
	Ban on national TV, radio and print media as well as on some but not all other forms of direct and/or indirect advertising
	Ban on all forms of direct and indirect advertising (or at least 90% of the population covered by complete subnational legislation)

4.1.2 Effect sizes of select interventions based on intensity level

The UN-interagency OneHealth Tool (OHT) provides effect sizes that correspond to each intensity level (**Table 3**).[20] These are "slices" of the full effect sizes reported in **Table 2** and are based on the opinion of WHO experts. For example, enacting advertising bans on national, television, radio, and print media only (light blue in Figure 2) is expected to result in a 0.6 percent absolute reduction in smoking prevalence, compared to three percent for enacting a ban on all forms of direct and indirect

[1] We assessed plain packaging on a 0, 1 scale— (i.e. it was either enacted by a country, or not).

advertising (dark blue)—i.e. a country is “missing out” on 2.4 percent (3.0% - 0.6% = 2.4%) of the potential impact of TAPs bans, because it has not yet moved the measure to its highest intensity level.

Table 3. Absolute Reduction in Smoking Prevalence by Intensity Level

Intervention	Complete absence	Level 2	Level 3	Full Intensity
Smoke-free air laws	0	0.44%	1.8%	7.0%
Graphic warning labels	0	1.0%	2.0%	4.0%
Mass media	0	0.92%	3.3%	6.0%
TAPS bans	0	0.6%	1.8%	3.0%

A country may regulate and implement a measure, but if the population and/or institutions are not compliant with the legislation it is likely to mitigate the intended effect of the policy. For example, if cultural norms dictate that smoking in public places is acceptable and/or enforcement of smoke-free public places is low people may smoke in restaurants or bars regardless of whether regulation and/or penalties (social, monetary, or otherwise) are nominally the law of the land.

The GTCR provides a qualitative assessment of compliance with two measures: smoke-free air laws and advertising, based on consultation with five national experts who assess compliance on a 0-10 scale. For these measures, we adjusted effect sizes to account for reported levels of compliance. Following previously published assumptions by Levy and colleagues (2013), we assumed that respectively 25 percent and 50 percent of the effect of these measures depends on levels of compliance.[21]

4.2 The impact of tax measures

The impact of cigarette tax increases on revenue and cigarette use prevalence was estimated using an Excel-based tool developed to analyze the impact of tax increases on a fixed population cohort. The tool was populated with data, including on current cigarette smoking prevalence, the tax structure and applied tax rates, cigarette prices, demand, prevalence, and income elasticities, and inflation and income projections.

Variously, investment cases examined increases in cigarette taxation strong enough to reach goals to achieve tax bases equivalent to at least 75% of the retail price of cigarettes and that outpaced inflation and income growth. The impact of tax increases on cigarette use prevalence is dependent on prevailing elasticities: the extent to which individuals change use of a product (e.g., decrease consumption or quit) because of changes in the price of a tobacco product. Changes were calculated following Joossens and colleague’s (2009), who use a log-log function to ensure large price increases do not result in implausible reductions in consumption or prevalence.[22] Below, **Equation 1** provides an example of calculations to ascertain the impact of a change in price on smoking prevalence, considering changes in income.

Equation 1. The impact of changes in price on smoking prevalence

$$\Delta SP_i = SP_{i-1} * ((EXP\left(\mathcal{E}p * LN(op\_np)\right)) - 1 - \left[\frac{1+ \in_i\left(\frac{GDP_2-GDP_1}{GDP_2+GDP_1}\right)}{1- \in_i\left(\frac{GDP_2-GDP_1}{GDP_2+GDP_1}\right)}\right]$$

Where:

SP = smoking prevalence (# of smokers) in year i

Ɛp = prevalence elasticity

Op_np = the ratio of the old price of a pack of cigarettes to the new price after tax increases

Ɛi = income elasticity

GDP = Gross domestic product in year (used as a proxy for wage growth)

There were several limitations to the tax analyses. First, the tool was only framed to assess the tax structure of the most sold brand of cigarettes and thus the analysis inherently assumes that those cigarettes are representative of the entire market (i.e., we did not assess other market segments—high or low-end cigarettes). More detailed models that account for switching between segments or between products (e.g., movement to hand-rolled cigarettes) would capture nuance helpful to framing tobacco tax policy and estimating impact. Second, the analysis assumed a full pass through the tax increases. This assumption reflects a "middle ground" approach, but the tobacco industry may increase or decrease prices in reaction to the price increase.

4.3 The impact of brief advice to quit tobacco use at the primary care level

We calculated the effect of scaling up the provision of brief advice to quit smoking at the primary care level. First, we calculated the baseline population quit rate (PQR, the percent of smokers who quit annually) drawing on previously published methods by Levy and colleagues (2010).[14] The PQR was calculated using three datapoints: quit attempts, treatment utilization rates (i.e. counselling, pharmaceutical therapy) and treatment effectiveness.

Equation 2. Calculating Population Quit Rate, from Levy et al (2010) (2)

$$PQR = QA * \sum_{i=1\ldots4} (TxUse_i * TxEff_i)$$

Where:

PQR = Population quit rate

QA = % of smokers who make a quit attempt at least once annually

TxUse = the percent of those who make a quit attempt who use treatment category i

TxEff = The percent of those who use a given treatment who succeed in quitting annually (Treatment efficacy)

i = is one of four treatment categories: 1) no evidence-based treatment; 2) counselling; 3) pharmacological treatment (e.g. nicotine replacement therapy), or 4) both counselling and pharmacological therapy.

Again following Levy et al (2010), "to account for the effect of multiple quit attempts among those who fail at their first attempt, it was assumed that half of those that make at least one quit attempt per year go on to make a second attempt, and half of those [who make a second attempt] make a third, and so on," and that treatment effectiveness does not change based on whether it is a persons' first quit attempt or a succeeding one.

After establishing baseline PQR, we calculated how the population quit rate would change if healthy lifestyle counselling was scaled at the primary care level. In this "intervention scenario", over the 15-year time horizon of the analysis, half of all primary health care providers are trained to provide brief advice to quit to adult tobacco users—a value selected based on evidence of the current intervention coverage gap; on average, in low- and middle-income countries less than half (47.8 percent) of adult smokers who visit a health provider are advised to quit.[2] Once trained, it was assumed that the provider administers the brief advice when they encounter a patient who uses tobacco.

Taking into account the number of primary healthcare providers in the country, the patient panel size per provider, adult smoking rates, and the percent of adult smokers who presented within the health system for at least one primary care visit per year, in each year of the analysis we calculate the number of adult tobacco users who would encounter a newly trained health provider and receive the brief intervention—which increases the likelihood that an individual makes a quit attempt by 60 percent over baseline levels.[14] With increases in population quit attempts driven by the provision of brief advice, we recalculate PQR to estimate the number of smokers who quit because of the intervention.

4.4 Combined Impact: Effect Size of Implementing Multiple Policies Simultaneously

The combined impact of all provisions together was given as:

$$ES = 1 - (1 - PR_i) * \left(1 - PR_j\right) * \ (1 - PR_k) \ldots$$

Where:

ES = the combined relative reduction of the analyzed tobacco-control provisions

PR = the constant relative reduction of policy i, j, k, …

4.4 Modelling the impact of the measures on health and socio-economic outcomes

The economic model is a "static" model. This meant that aside from smoking prevalence reductions catalyzed by implementation of new tobacco control measures, other variables did not change throughout the time horizon of the analysis (e.g., the population did not grow, or change in size or makeup (age/gender).

Forecasted changes in smoking prevalence were applied directly to outcomes health and socio-economic outcomes assessed in the cost-of-illness analysis. This meant, for example, that we adjusted the risk factor attributable outcomes for mortality and morbidity as reported by GBD based on year-over-year relative changes in smoking prevalence for each outcome (i.e., if prevalence dropped, in relative terms, by 20 percent so too did smoking-attributable mortality and morbidity). For simplicity, the model assumed these changes to be immediate (i.e., it did not account for lag time of disease onset).

To calculate the impact of measures, we subtracted the outcome (risk factor attributable deaths, healthcare expenditures, etc.) under the intervention scenario from the same outcome under the base

[2] Analysts pulled data from GATS surveys conducted between 2009 to 2018 and averaged values from low- and middle-income countries.

scenario. The difference between the two outcomes represents the reduction in burden, or savings in terms of lives or costs, that could be achieved by the tobacco control measures.

$$Marginal\ Effects = Outcome_{Base\ Scenario} - Outcome_{Intervention\ Scenario}$$

5. The Financial Cost of Implementing and Enforcing Tobacco Control Measures

The financial costs to the government of implementing new measures—or of intensifying or enforcing existing ones—was estimated using the WHO NCD Costing Tool. Full explanations of the costs and assumptions embedded in the WHO NCD Costing tool are available.

The Tool uses a 'bottom up' or 'ingredients-based' approach. In this method, each resource that is required to implement the tobacco control measure is identified, quantified, and valued. The Tool estimates the cost of surveillance, human resources, trainings and meetings, mass media, supplies and equipment, and other components. Within the Tool, costs accrue differently during five distinct implementation phases: planning (year 1), development (year 2), partial implementation (years 3-5), full implementation (years 6 onward).

Across these categories, the Tool contains default costs from 2011, which are sourced from the WHO CHOICE costing study. Following Shang and colleagues, the Tool was updated to reflect current costs by updating several parameters: the USD to LCU exchange rate, purchasing power parity (PPP) exchange rate, GDP per capita (USD), GDP per capital (PPP), population (total, and share of the population age 15+), labor force participation rate, and government spending on health as a percent of total health spending.[23]

The WHO NCD Costing Tool tobacco control module contains estimates for the following policies and programs: program strategy development, smoke-free policies, tobacco taxes, package warnings, advertising bans, cessation programs, and media campaigns. Because the Tool does not contain estimates for implementing plain packaging, we assumed the cost of this intervention to be equal to that of implementing warning labels since they both involve regulation of the packaging of cigarette packaging. For the calculation of return on investment for the package of interventions, the cost of program strategy development is included in the total cost. If countries had already fully implemented one of the measures, it was not included as a cost in the analysis.

6. Return on Investment (ROI)

Return on investment is a measure of efficiency, weighing the monetized socio-economic outcomes generated by tobacco control measures against the financial costs of their implementation. ROI was calculated as:

$$\textbf{Return on Investment (ROI)} = \frac{\text{Benefits of Intervention/Policy}}{\text{Costs of Implementing Intervention/Policy}}$$

7. References

1. World Health Organization, *Global Health Observatory data repository*. 2014.
2. Institute for Health Metrics and Evaluation. *Global Burden of Disease Results Tool, 2019*. 2020 2020 January 2021]; Available from: http://ghdx.healthdata.org/gbd-results-tool.
3. International Labour Organization, *Mean nominal monthly earnings of employees by sex and economic activity -- Harmonized series, Nov. 2019 (5) - Annual | Mean real monthly earnings of employees, annual growth -- ILO modelled estimates, Nov. 2019 (5) - Annual.* 2019: ILOSTAT database.
4. Haacker, M., T.B. Hallett, and R. Atun, *On discount rates for economic evaluations in global health.* Health Policy Plan, 2020. **35**(1): p. 107-114.
5. International Labour Organization, *Employment-to-population ratio by sex and age -- ILO modelled estimates, Nov. 2019*. 2019: ILOSTAT database.
6. The World Bank, *World Bank Open Data*, The World Bank, Editor. n.d.
7. World Health Organization, *Global Health Expenditures Database - 2018 expenditures*. 2020: online.
8. Goodchild, M., N. Nargis, and E. Tursan d'Espaignet, *Global economic cost of smoking-attributable diseases.* Tob Control, 2018. **27**(1): p. 58-64.
9. Robinson, L., Hammitt, J., Cecchini, M., Chalkidou, K., Claxton, K., Cropper, M., Eozenou, P., Ferranti, D., Deolalikar, A., Guanais, F., Jamison, D., Kwon, S., Lauer, J., O'Keeffe, L., Walker, D., Whittington, D., Wilkinson, T., Wilson, D., Wong, B., *Reference Case Guidelines for Benefit-Cost Analysis in Global Health and Development*. 2019, Bill & Melinda Gates Foundation.
10. Agriculture, U.S.D.o., *Value of statistical life year (VSL) in the United States*. 2019.
11. World Health Organization, *WHO report on the global tobacco epidemic, 2021*. 2021: Geneva, Switzerland.
12. World Health Organization, *WHO NCD Costing Tool*. 2010: Online. p. Tools for implementing WHO PEN (Package of essential noncommunicable disease interventions).
13. Levy, D.T., et al., *The Impact of Implementing Tobacco Control Policies: The 2017 Tobacco Control Policy Scorecard.* J Public Health Manag Pract, 2018.
14. Organization, W.H., *Tobacco control interventions*, in *Technical briefing for Appendix 3 of the Global Action Plan for Non-Communicable Diseases*. 2018.
15. Nargis, N., et al., *Price, Income, and Affordability as the Determinants of Tobacco Consumption: A Practitioner's Guide to Tobacco Taxation.* Nicotine Tob Res, 2021. **23**(1): p. 40-47.
16. Levy, D.T., et al., *Modeling the impact of smoking-cessation treatment policies on quit rates.* Am J Prev Med, 2010. **38**(3 Suppl): p. S364-72.
17. Troelstra, S., et al., *Smoking and sickness absence: a systematic review and meta-analysis*. 2020, Scandinavian Journal of Environmental Health.

Nugent R, *et al. Tob Control* 2024; 33:10–16. doi: 10.1136/tc-2023-058336

18. Baker, C.L., et al., *Benefits of quitting smoking on work productivity and activity impairment in the United States, the European Union and China*, in *Int J Clin Pract*. 2017.

19. Berman, M., et al., *Estimating the cost of a smoking employee.* Tob Control, 2014. **23**(5): p. 428-33.

20. World Health Organization. *OneHealth Tool*. Cost effectiveness and strategic planning (WHO-CHOICE) [cited 2018 Feb 24]; Available from: http://www.who.int/choice/onehealthtool.

21. Levy, D., et al., *Smoking-related deaths averted due to three years of policy progress*. 2013, World Health Organization: Bulletin of the World Health Organization. p. 509–518.

22. Joossens, L., et al., *How Eliminating the Global Illicit Cigarette Trade Would Increase Tax Revenue and Save Lives*. 2009, Bloomberg Philanthropies and the Bill and Melinda Gates Foundation: online.

23. Shang, C., et al., *Country-specific costs of implementing the WHO FCTC tobacco control policies and potential financing sources.* PLoS One, 2018. **13**(10): p. e0204903.
