# Supplementary material for: Evolving methodology of national tobacco control investment cases
Source: Tob Control. 2024 May 2;33(Suppl 1):s10–6. doi: 10.1136/tc-2023-058336 (PMC11103330; doi:10.1136/tc-2023-058336)
Supplement: Supplementary data [file tc-2023-058336supp002.pdf]

Supplemental File 2. Diseases causally associated with tobacco use

Tuberculosis
Other Cardiovascular and Circulatory Diseases
Lower Respiratory Infections
Lip and Oral Cavity Cancer
Nasopharynx Cancer
Esophageal Cancer
Stomach Cancer
Colon and Rectum Cancer
Liver Cancer
Pancreatic Cancer
Larynx Cancer
Tracheal, Bronchus, and Lung Cancer
Cervical Cancer
Kidney Cancer
Bladder Cancer
Leukemia
Interstitial Lung Disease
Diabetes Mellitus
Ischemic Stroke
Other Chronic Respiratory Diseases
Cataract
Macular Degeneration
Hypertensive Heart Disease
Ischemic Heart Disease
Atrial Fibrillation and Flutter
Hemorrhagic Stroke
Peripheral Artery Disease
Aortic Aneurysm
Chronic Obstructive Pulmonary Disease
Asthma
Coal Worker’s Pneumoconiosis
Asbestosis
Silicosis
Other Pneumoconiosis
Peptic Ulcer Disease
Rheumatoid Arthritis
Non-Hip Fracture
Hip Fracture
